# Effect of the Ripening Period and Intravarietal Comparison on Chemical, Textural and Sensorial Characteristics of Palmero (PDO) Goat Cheese

**DOI:** 10.3390/ani11010058

**Published:** 2020-12-31

**Authors:** Sergio Álvarez, María Fresno

**Affiliations:** Unit for Animal, Grassland and Forage Production in Arid and Subtropical Areas, Canarian Institute of Agrarian Research, 38270 San Cristóbal de La Laguna, Spain; mfresno@icia.es

**Keywords:** goat cheese, sensorial characteristics, colour, texture, ripeningprocess

## Abstract

**Simple Summary:**

Palmero PDO (Protected Denomination of Origin) cheese is a typical product of La Palma (Canary Isles, Spain) and it is manufactured from raw goat milk of the Palmera breed. All goat herds must be fed with local vegetal resources: pastures and or/grazing. It is an uncooked, pressed cheese, commercialised both fresh (from 8 to 20 days), as semi-hard (from 21 to 60 days) and hard (from 60 days). The aim of this study was to evaluate the changes in the physicochemical and sensorial parameters of Palmero PDO cheeses during 90 days of aging, also making an intravarietal comparison between dairy plants. This characterization could lead a better and complete cheese definition. Some variations have been observed between cheese artisanal factories because each cheesemaker has some cheese making particularities that are inherited from parents to children. These differences can be used for purchasing and marketing as added values linked to “terroir” and cheese handmade practices.

**Abstract:**

Palmero cheese is an artisanal dairy product from the Canary Islands (Spain), awarded with the Protected Denomination of Origin (PDO) from the European Union. It is made with raw milk from the Palmera dairy goat on La Palma island. The aim of this research covered the physicochemical and sensorial characterization of Palmero cheese along 90 days of ripening. Palmero cheeses from four cheese factories were analysed for basic physicochemical parameters, instrumental texture and colour and sensorial profile. Most of the basic composition and the texture and colour attributes of Palmero cheese changed significantly along maturation. During the 90 days of ripening an increase in hardness, fracturability and gumminess (*p* < 0.001) occurred while elasticity decreased simultaneously (*p* < 0.001). The internal lightness value decreased significantly (*p* < 0.001), while yellowness increased (*p* < 0.001) during cheese ripening. Ripening time affected six of nine sensorial texture characteristics and the entire odour and flavour parameters analysed (*p* < 0.001). Regarding to intravarietal comparison, in general, cheeses from the four dairy plants showed similar composition although significant differences were detected on textural, colour and sensorial characteristics.

## 1. Introduction

Food quality is a complex notion that includes several aspects: legal, nutritional, hygienic and sensorial [1]. Nowadays, there is a great deal of interest in the definition of quality, especially with regard to the Protected Denomination of Origin (PDO). These products are manufactured in a very traditional way, with differences between the texture, smell, aroma and taste of the cheeses depending on the producers [2].Therefore, it is of great interest to study the characterization of these products throughout their maturation process, in the different stages of their commercialization Palmero cheese is a typical product of La Palma (Canary Isles, Spain) and it is manufactured from the raw goat milk of the “Palmera” breed according to the specifications of its Denomination of Origin Regulatory Board (EU 1241/2002) [3]. This indication implies that this product has distinctive sensory characteristics which should be connected to the traditional production methods, guaranteeing the consumer a specific sensory quality [4].

This cheese is not only a PDO product but also meets the specific characteristics of a “terroir product” by its specific attributes linked to natural factors as soil, climate or natural vegetal resources and by human factors related to history, culture know-how and tradition [5]. There are two varieties of Palmero Cheese, raw milk cheeses and pasteurised ones, although nowadays all cheesemakers only uses raw milk. It is an uncooked, pressed cheese, commercialised both fresh (from 8 to 20 days), as semi-hard (from 21 to 60 days) and hard (from 60 days). An authentic Palmero Cheese must have cylindrical shape, with flat sides and must keep certain proportions: 6–15 cm high and 12–60 cm in diameter. Currently, 30 cheesemakers meet the requirements of the Protected Designation of Origin Palmero Cheese. All herds must be fed with local vegetal resources: pastures and/or grazing. These products should have “added value” for the consumer with regard to: health benefits of dairy fat, authentication, food and environmental sustainability, animal-welfare, local origin and linked to terroir [6].

Cheese composition is mainly controlled by the initial composition of the cheese milk and the manufacturing protocols (e.g., pH at renneting and draining, size of curd particles, temperature, method of salting) used for cheese making. Texture and colour are important criteria for evaluation of cheese quality as these two parameters are important for consumers in making decisions on the purchase of the product [7]. The exact role that texture plays with consumer acceptance is difficult to define because flavour and visual appearance cannot be uncoupled from texture when the consumer evaluates cheese [8]. Additionally, colour is one of the first quality attributes that a consumer uses to judge the acceptability of a product [9]. The physical properties of cheese (i.e., texture and colour) are influenced by initial cheese milk composition, manufacturing procedures, and maturation conditions [10]. Many of the major changes in cheese structure, which ultimately affects final texture, occur during storage [11]. Rheological procedures define the physical characteristics of cheese, although they must be compared and used joint with tactile and visual human perceptions in order to understand cheese texture properties [11].

Cheese ripening involves a very complex series of interrelated events, converting the fresh curd to a specific cheese variety with a determined appearance, texture, odor, flavour and taste characteristics [12]. All of these are involved in the definition of cheese quality and are related to the intensity of the ripening process in terms of proteolysis, lipolysis, and glycolysis [13].

Texture performs a fundamental role in consumer’s evaluation, sometimes being even more important than external, taste or smell characteristics. In addition, it is an indicator of quality, related to how the brain processes physical features during the chewing process [14]. On the other hand, colour is one of the main characteristics which defines the quality of the product, and according to Calvo [15], is the one that most influences the consumer’s choice. Out of all the sensory qualities presented by a food, colour sometimes can even subjectively modify other sensory perceptions such as odour and flavour.

The sensory quality of food products comprises different organoleptic primary attributes as visual appearance, texture, odour, aroma and taste. These types of attributes are determinative characteristics in the election of foodstuffs by the consumer. Sensory analysis is clearly the most valid means of measuring flavour characteristics [16]. This type of analysis is necessary to describe sensory properties of cheese and consumer acceptability [17]. Odour and flavour descriptors in goat cheeses are associated with particular characteristics of goat milk, such as the lipolytic system, and specific fatty acids and their evolution during maturation. Various volatile compounds, such as free fatty acids, deeply influence typical goat flavour [18].

Physicochemical and sensorial characteristics of cheeses made with Palmera goat milk in experimental conditions have been evaluated in different research studies [19,20] and Palmero PDO cheese characteristics have been partially described before [21,22,23,24] but there is no in-depth study of the major physicochemical and sensory characteristics along maturation. Furthermore, to date, an intravarietal comparison has not been investigated.

This paper is written as a part of the report for the strategic project of the Canary Government for increasing the characterization and differentiation of Canarian PDO cheeses, including studies of changes that occur during ripening and consumer preference evaluations. The aim of this work was to describe changes in the physicochemical and sensorial characteristics of the Palmero cheese during ripening, including an intravarietal comparison from four different dairy plants.

## 2. Materials and Methods

### 2.1. Sampling and Cheese-Making Procedure

With the agreement of a panel of professionals (veterinarians, farmers, cheese makers, and marketing agents), 4 artisanal cheese producers were selected for their quality and consistency of Palmero cheese production adhering to PDO regulations [3].

Cheeses were manufactured from raw milk following the specifications of the Palmero Cheese Denomination of Origin Regulatory Board (2002) [3]. A total of 48 Palmero goat cheeses were manufactured (12 from each cheesemaker) the same day as milking, using Palmero goats’ milk with a minimum of 3.80%, 4.00% and 12.50% of protein, fat and total solids, respectively. The milk was coagulated with animal rennet from kid´s abomasum, at a temperature between 27 and 33 °C for approximately 45 min. Curds were subsequently cut to obtain grains of less 3 mm in diameter with a previous press in the batch to eliminate part of the whey. The curd was then moulded into pieces of approximately 2 kg. Afterward, salting with dry sea salt was achieved by rubbing dry salt onto the surface of the cheeses. The cheeses were ripened in the same ripening chamber located in PDO Palmero Council with controlled ripening factors at 10 to 12 °C and 85% to 87% relative humidity. Although it is not a usual practice, the producers of Palmero PDO cheese ripen their cheeses in a collective ripening chamber supervised by the technicians of the PDO regulatory council. From each cheese factory, 3 cheeses were picked up after 15, 30, 60 and 90 days of ripening to study physicochemical and sensorial characteristics (four cheese factories × three cheeses × four ripening periods). These periods were selected by the PDO technicians as the most suitable from a commercial point of view. Cheese samples were coded with a letter representing the respective dairy plant where they were manufactured, and a sample ID number was assigned to all cheese samples. For each aging time, cheeses were sent to the laboratory in refrigerated boxes and analysed immediately to evaluate the changes.

### 2.2. Physicochemical Analysis

Chemical cheese analyses were made in triplicate using a near-infrared spectroscopy (Instalab 600, Foss Electric, Slangerupgad, Denmark), calibrated for total solids content by ISO 5534:2004, fat content by ISO 11870:2009 and nitrogen content by ISO 8968-1:2014.Besides, cheese pH was determined at 20 °C using a pH meter InoLab Level 1 from WTW (Weilheim, Germany).The pH of cheese exterior was measured at a depth of 1 cm from the outer surface, while internal pH was measured in the center of the cheese.

### 2.3. Texture and Colour Measurements

Texture characteristics were determined using a Texture Expert Exceed XT2i (Surrey, England) by carrying out a texture profile analysis (TPA) as is described in detail by Fresno and Álvarez [25]. Six parameters were obtained for a double compression: hardness (N), fracturability (N), adhesiveness (N.s), cohesiveness, elasticity (%) and gumminess (N).

Internal and external colour was determined using a portable Minolta spectrocolourimeter (Minolta CR-400, Osaka, Japan) following the guidelines previously described by Fresno and Álvarez [25]. Five colour parameters were determined according to the CIELCH and CIELAB colour space: L*, chroma, hue angle, a*, and b*.

### 2.4. Sensory Analysis

Sensory analysis was carried out after 15, 30, 60 and and 90 days of ripening. Samples, coded with 3-digit random codes, were presented balanced to avoid the effect of the presentation order. The methodology employed has been previously described, with odour and flavour attributes in accordance with those described by Berodier et al. [26], and texture following the guidelines published by Lavanchy et al. [27]. This methodology has been adapted to goat cheeses by Fresno et al. [28]. A panel of seven formally trained and highly experienced judges was used, who already work in collaboration with the Palmero PDO cheese sensory panel. Furthermore, before the beginning of this experiment, five extra training sessions with Palmero PDO cheeses were performed.

The sensorial evaluation was carried out in a specific room for sensory analysis in the Canarian Institute of Agrarian Research (ICIA) [29], following the methodology formerly explained in Álvarez et al [20]. Each judge received two portions per sample of cheese, one for texture and the other for odour and flavour. Using a structured scale from 0 to 7, 9 parameters for texture, and 6 attributes for odour and flavour, were determined. In addition, each judge described specific descriptors for odour and flavour.

### 2.5. Statistical Analysis

SPSS version 15.0 (SPSS Inc., Chicago, IL, USA) was the package used for statistical processing of the results. A General Linear Model (GLM), MANOVA, was used to establish statistical differences in the values of the physicochemical parameters and the scores of the sensory analyses according to the maturation time and the comparison of the cheese factory. Post hoc multiple analyses by Tukey’s test and multiple regression were performed for ripening time factor and intravarietal comparison. Principal component analysis (PCA) was performed for varietal factor and discriminant analysis was performed for ripening time factor. Pearson correlations were undertaken between physicochemical and textural variables. The linearity of the relationships was graphically checked.

## 3. Results and Discussion

### 3.1. Basic Physicochemical Characteristics

The least square means for physical and chemical composition together with ANOVA results are presented in Table 1. Ripening affected all physicochemical characteristics (*p* < 0.01). Internal pH and fat content increased significantly along 90 days of ripening while moisture decreased. The external pH decreased from 6.50 to less than 5 points in the first 15 days after production (data not shown), afterwards it was maintained for up to 60 days of ripening and finally increased significantly. The pH of the cheese increased during ripening as a consequence of the consumption of lactic acid and the alkalizing effect of the compounds generated during protein degradation [30]. The cheese acidity level has great importance, influencing the growth of micro-organisms and enzymatic activity throughout the maturation process, as well as affecting the rheological properties and flavour [31,32].

Palmero pH cheese values fluctuated between 4.90–5.36 during the ripening period. The final average pH value (5.17–5.36) is close to the range of values observed by different authors for other Canarian PDO goat cheeses [25]. The role of pH in cheese texture is particularly important because changes in pH are related directly to chemical changes in the protein network of the cheese [7]. The surface pH value is higher than the internal one, as observed by Fresno et al. [33] when studying the Armada cheese variety. As regards the moisture content, the highest value was measured at 15 days, after which it decreased significantly till 60 days while remaining constant up to 90 days. Fresno and Alvarez [25] obtained a similar moisture behaviour in Majorero cheeses (another Canarian goat PDO cheese) although the Palmero values were considerably lower. Cheese moisture is controlled by the velocity and extent of syneresis and the contraction of the casein structure. In moulding, the pressing and salting processes, which follow coagulation, the decrease in pH plays an important role resulting in a significant whey diminution [34]. The protein concentration showed irregular values but with small fluctuation, only 4 percentage points between the lowest value (30 days) and the highest value (60 days). These results are similar, although slightly lower, than those determined by other authors for cheeses made with local breed milk [25,33]. The total fat content of the Palmero cheese at the start of ripening was 47.72%; this significantly increased to 52.43% (*p* < 0.01) at 60 days, and then remained constant till the end of the maturation period. The protein and fat contents observed in Palmero cheese during ripening (expressed as g/100 g of TS) showed values of around 30 and 50, respectively. These fat statistics are in the range of values observed for other Spanish goat’s milk cheese types [25,33] but are quite lower compared to other Palmero cheese protein concentrations [19].

### 3.2. Texture

Table 1 also shows the values for textural attributes for Palmero PDO cheeses derived from TPA analysis. Texture profile analysis is an important tool in cheese characterization. All textural parameters of the cheeses were affected by ripening time (*p* < 0.001), except cohesiveness that presented similar values for all different ripening periods. These results are in accordance with those reported by other authors [25,35,36]. Fracturability and gumminess increased along maturation, as well as fat raised [37]. However, Pinho et al. [7], studying “Terrincho” ewe’s cheese, recorded an increase in these parameters up to 30 days and then a decrease afterwards to the end of the maturation. Although Palmero PDO cheese textural behaviour is similar to Majorero PDO cheese [12], fracturability, hardness and gumminess values are considerably higher, due to the different technologies applied. The most important factor could be the intensive cut until grains are less than 3 mm in diameter. An increase of 110% in the hardness values has been found at 90 days of ripening when compared with 15-day old cheese. A direct correlation has been detected between hardness and moisture, this correlation has also been noted by Pompei et al. [38] and Tejada et al. [39]. Decreased water content promotes a greater casein concentration and an increase in the number of casein bonds; both factors increased hardness values. Moreover, water plays a plasticizer role between the protein molecules, making the cheese softer [40]. Similar hardness value evolution was determined by Fresno et al. [19] on Palmero experimental cheeses made with different types of coagulants.

On the other hand, elasticity, which is the degree of recovery of a deformed piece of cheese after the deforming force is removed [41], decreased till 30 days ripening and remained constant till 90 days, while adhesiveness showed irregular fluctuations, decreasing in the first two months of maturation and increasing thereafter. These results could be related to the increasing fat content in the ripening process of the cheeses [42,43]. The fat content increment modifies the texture properties. These changes in texture are characterised by increased elasticity, decreased cohesiveness and decreased smoothness of mass [44]. Lawence et al. [45] reported that the pH of cheese had a large effect on textural properties. A direct correlation between fracturability, hardness (*p* < 0.001) and gumminess (*p* < 0.01) with pH has been detected. The observed increases in rheological fracturability, hardness and gumminess can be attributed to the dissociation of calcium phosphate bridges between casein molecules with increasing pH. Decreasing pH towards 5.4 allows greater hydrophobic interaction between the protein molecules and causes the curd to become firmer and more elastic. Everett and Olson [46] found that strain and fracture increased as the pH increased from 5.0 to 5.25 in Cheddar cheese. For Palmero cheese, pH increased from 4.90 to 5.37 till 90 days of ripening, allowing a firmer, elastic and crumbly curd. Oppositely, Watkinson et al. [31] found that increasing pH resulted in less crumbly and firmer cheeses. Fracturability and hardness, especially due to its high correlation with moisture, fat and protein content, and also gumminess could be used as interesting ripening predictors.

### 3.3. Colour

The mean values for L*, chroma, hue angle, a* and b* parameters are shown in Table 1. Cheese colour was statistically affected (*p* < 0.05) by ripening time. Only external chroma and b* and internal a* and hue angle were not affected by this factor. Both external and internal lightness decreased along maturation according to Rohm and Jaros [47]; this trend was more prominent on the surface than inside the cheese. As was referred for certain textural parameters, lightness could be an appropriate parameter for ripening prediction. The 30- and 60-days aged cheeses showed higher internal colour intensity while external colour tone was significantly (*p* < 0.05) higher in fresh cheeses (15 days). The increase in colour intensity with ripening time has already been reported in other cheese varieties, such as Cheddar [48], Mahón [49], Emmental [50], and Los Pedroches [39]. An indirect correlation (*p* < 0.05) was detected between moisture and colour, a finding also noted by Rohm and Jaros [47] and Frau et al. [49] in cow cheeses, and Tejada et al. [39] in experimental ewe cheeses made with different coagulants.An increase in yellowness b*(i) was observed up to 60 days of ripening; however, cheeses became less yellow at 90 days. Palmero PDO cheeses showed less L* and chroma values than Palmero experimental cheeses [19]. As was observed by other authors [7,50], there was a decrease in lightness and a slight increase in both redness (a) and yellowness (b) during cheese ripening in the present study.

### 3.4. Sensorial Analysis

The results of the sensory evaluation of Palmero cheeses are shown in Table 2 for texture attributes. Ripening time affected six of nine texture characteristics. Moisture (superficial and mouth) decreased (*p* < 0.001) throughout the maturation period, obtaining the lowest values at ninety days. The humidity loss is frequently associated with lower dough elasticity [51]. It has been suggested that higher moisture content allows a greater movement of the casein matrix and reduces resistance to deformation in hard cheeses [52]. Moisture content has been reported to influence the fracture mechanism during biting and mastication [53]. As was expected, superficial and mouth moisture were directly correlated with physicochemical moisture and indirectly correlated with protein and fat contents (Table 3).

Roughness and friability increased until 60 days. The values stabilized in the last thirty days. Older cheeses were less adhesive than fresh ones. Adhesive parameter decreases along ripening, not being related with fat increment, in contrast with other cow [54] and experimental cheeses [43]. In addition, the firmness, solubility and granulosity values were very similar throughout the ripening period. These results are in accordance with Majorero [55] and Cheddar cheeses [56] although other studies recorded an increase for older cheeses [57,58].

Ripening time affected all odour and flavour parameters analysed (Table 2). The odour and flavour intensity increased significantly (*p* < 0.001) during ripening, as was also recorded by Agabriel et al. [54]. A general increase in flavour is perceived in the ripening process, this increment with age is caused by the production of a wide range of volatile compounds during maturation by the metabolism of triglycerides and proteins [59]. In other cheeses, the intensity of the sensory attributes was found to increase with ripening time, even though the increase was not significant for all attributes [60]. Odour and flavour of cheese results from the correct balance and concentration of numerous sapid and aromatic compounds perceived during cheese consumption [13]. Along maturation, proteolysis and lipolysis increase, promoting the appearance of certain volatile compounds responsible for the odour and flavour characteristics [39,61]. These compounds have already been detected in goat milk cheese varieties [62,63].

Fresh cheeses presented lactic odours, mainly associated with goat raw milk, and citric aromas, commonly lemon, while older cheeses changed into more complex descriptors such as butter and hay odours, although lactic aromas were still evident. Compared with another Canarian cheese, Majorero PDO cheese [25], Palmero cheese showed higher friability, firmness and granulosity scores, while it was less humid and soluble. Trigeminal stimulation presented an unequal behaviour during ripening. While saltiness and pungency values increased as was described by Engel et al. [64], bitterness decreased up to 90 days and acidity up to 60 days, increasing afterwards. In Cantal cheese [54], saltiness, bitterness and pungency increase along maturation, while bitterness characteristics were previously observed by Tejada et al. [65] in Murcia al Vino cheese made with animal rennet. During maturation, due to the breakdown of the protein network bitterness increases due to bitter peptide formation [66]. Contrary to this, in our experiment bitterness values were higher at 15- and 30-days stages of maturation. The evolution of the acid content overlaps with the reports made by Gaborit et al. [62] in several goat milk cheeses. Finally, sweetness or astringent stimulation did not appear at all.

### 3.5. Relationships between Sensory and Rheological Parameters

Cheese texture is a critical quality attribute. Sensory texture is determined by descriptive analysis and instrumental texture is determined by rheological and fracture testing. In Table 3 and Table 4 correlation analysis results are shown. As was expected, superficial and mouth moisture were directly correlated with physicochemical moisture and indirectly correlated with protein and fat contents. Hardness was correlated significantly with the sensorial parameters roughness, granulosity and friability and had a negative correlation with superficial moisture, elasticity and mouth moisture. Fracturability had the same correlations as hardness but with less friability interaction. Although Foegeding and Drake [8] have reported clear correlations between sensory and mechanical measures of hardness and firmness, these two parameters were not correlated in Palmero cheeses. None of the sensorial parameters correlated significantly with cohesiveness or adhesiveness. These results are in agreement with Foegeding and Drake’s [8] studies, where chewdown sensory terms that measure adhesiveness, cohesiveness and gumminess were poorly correlated with mechanical parameters. Furthermore, elasticity was correlated with superficial humidity, a parameter which in turn was correlated with cheese moisture.

### 3.6. Intravarietal Comparison

The physicochemical composition, the texture attributes of TPA and the colour parameters of the 12 samples of Palmero cheeses with 30 days of ripening from four different dairy plants are shown in Table 5. This 30 day ripening period was chosen for representing the highest consumption stage of Palmero cheese. All the values of the parameters analysed remain within the ranges of the Palmero PDO Board [3]. Although all PDO cheeses must maintain similar physicochemical and sensory characteristics within a specific range of variability, each cheesemaker can slightly modify different technological aspects, both in cheese practices and, for example, influencing the maturation pattern by controlling the temperature and the humidity of the environment.

In general, cheeses from the four dairy plants presented similar compositions except for internal pH that showed moderate variability between cheesemakers (*p* < 0.01) fluctuating between 4.82 to 5.10. External pH, fat and protein content and moisture percentage presented small variability. This fact could be due to the similar physicochemical characteristics of the milks used and the similar feeding system (pasture and grazing). Regarding texture attributes of TPA, cheeses from B and C dairy plants presented similarity with respect to fracturability, elasticity and gumminess, while A and D factories showed similar results for cohesiveness, elasticity and gumminess. Hardness (*p* < 0.349) was the unique attribute that did not show variability between cheeses from different dairy plants. With respect to colour attributes, no significant differences were observed on L* measured on the exterior and the interior surface of the cheeses. Cheeses from plant C presented the greatest colour intensity and yellow percentage for external measures. These results were not repeated for internal measures. Although significant differences are perceived in most colour parameters, the determined values remain, however, in a narrow range of variability.

The sensorial attributes of Palmero goat cheese after 30 days of ripening are given in Table 6. Cheeses from plant A presented greater variability for organoleptic characteristics. This type of cheese was more firm, crumbly, salty and pungent while C cheeses were more acidic and soluble showing higher superficial and mouth moisture values. Cheese from the four dairy plants presented similar values for adhesivity. With respect to odour and flavour intensity, cheeses from cheese factory B presented the lowest values while dairy plant A showed the highest scores. It is important to note that all the sensorial values detected were within the ranges determined by the official sensorial panel of Palmero PDO cheese.

Palmero PDO cheese is a local artisanal production; even as their cheeses are made under PDO Regulatory Board conditions [3], they are difficult to standardize. All are handmade producers with know-how from parents to children, so all of them use an old secret formula that makes cheeses different. These variations in sensory attributes can add extra value within the quality and origin guaranty of the PDO.

Finally, factor analysis, using the principal components method for the extraction of factors, was applied to all the samples of cheeses studied from different dairy plants, to obtain a more simplified view of the relationship among the sensorial parameters analysed. It was considered more interesting to carry out the PCA analysis only with sensory descriptors, without including the instruments both for the knowledge and description of Palmero cheeses and for the possibility of using these results by producers and technicians. Because their Eigen values were higher than one, four factors were chosen (88.9% of the total variance), and therefore, they explain more variance than the original variables (Table 7). A Varimax rotation was carried out to minimize the number of variables that influence each factor and then facilitate the interpretation of the results. The first factor that explains the higher percentage (45.74%) is associated with the intensity of flavour and odour and with textural attributes such as friability and granulosity. Superficial and mouth moisture and acidity had the highest loadings on the second component, accounting for 22.75% of the total variance. The third component, explaining 13.07% of the variance, was defined mainly by saltiness and acidity.

**Figure 1:**
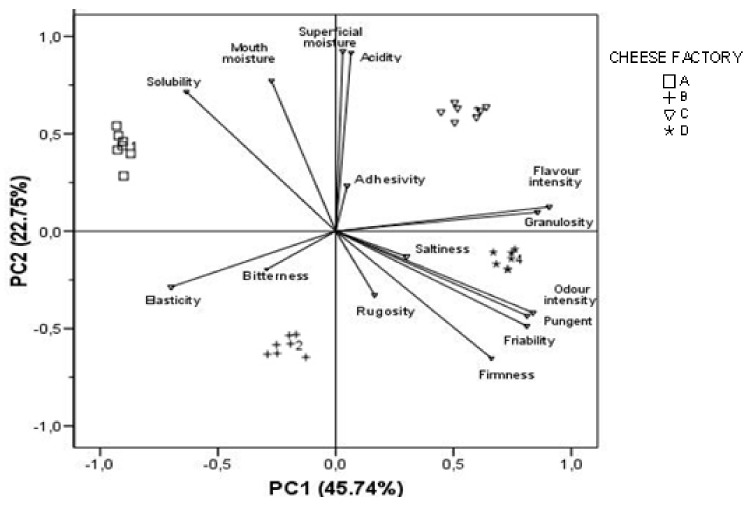
Principal component analysis of the sensory data. Correlation plot with distribution of cheese samples.

The results of the PCA have been depicted on a two-dimensional plot (Figure 1) that explained 68.5% of the total variance. The negative segment of the plot for PC1 was related to three sensorial texture parameters, elasticity, solubility and mouth moisture, and also bitterness as basic taste, whereas the positive segment of the plot for that factor was mainly related to odour and flavour intensity, granulosity, firmness and friability as texture characteristics, in addition to pungency stimulation. Moreover, the distribution of cheese samples is also presented where a high discrimination is registered between the 30-day cheese samples from the four dairy plants. Although the four plants are moderately separated, cheese factories C and D remain closer than A and B.

Finally, the stepwise discriminant analysis of all cheese samples according to ripening period (Table 8) showed that cheeses with different ripening stages were very well differentiated. Both 60- and 90-day cheeses are well classified; no cheeses were grouped in different groups. The 15- and 30-day cheeses are less well classified but a high percentage are still well grouped (92.9 and 82.1, respectively).

## 4. Conclusions

These physical results join together with chemical composition and sensorial properties will be used for a better and more complete definition of Palmero PDOcheeses at different ripening periods. The variation observed in texture and colour parameter results could be useful to estimate the ripening time, representing an objective method. Furthermore, some differences in sensorial profile have been detected between cheese artisanal factories. These differences showed that Palmero PDO cheese is not a standardised cheese, it is an artisan cheese. Differences in the sensory profile can be considered an advantage since these cheeses can satisfy the requirements of consumers with different tastes.

## Figures and Tables

**Table 1:** Effects of ripening on physicochemical, instrumental texture and colour parameters.

	Ripening Time				
	15 d	30 d	60 d	90 d	
		LSM			Effect
pH (external)	4.92 ± 1.10 ^a^	5.14 ± 1.11 ^b^	5.22 ± 1.02 ^b^	5.37 ± 0.10 ^c^	0.001
pH ( internal)	4.90 ± 0.12 ^a^	4.98 ± 0.14 ^a^	5.01 ± 0.14 ^a^	5.17 ± 0.18 ^b^	0.001
Moisture, %	36.93 ± 1.84 ^c^	33.50 ± 3.41 ^b^	28.19 ± 2.64 ^a^	29.20 ± 0.87 ^a^	0.001
Fat, % of TS	47.72 ± 3.54 ^a^	50.06 ± 3.45 ^ab^	52.43 ± 2.96 ^b^	51.34 ± 1.72 ^b^	0.003
Protein, % of TS	29.85 ± 0.88 ^b^	30.84 ± 1.74 ^b^	26.98 ± 1.30 ^a^	31.37 ± 1.19 ^c^	0.001
Fracturability	80.8 ± 17.60 ^a^	99.2 ± 24.06 ^b^	112.7 ± 29.71 ^c^	155.5 ± 23.09 ^d^	0.001
Hardness	125.0 ± 24.43 ^a^	163.1 ± 32.05 ^b^	197.3 ± 48.57 ^c^	262.9 ± 42.93 ^d^	0.001
Cohesiveness	0.10 ± 0.02	0.11 ± 0.3	0.11 ± 0.02	0.11 ± 0.02	0.104
Adhesiveness	1.95 ± 1.59 ^b^	1.77 ± 1.62 ^ab^	1.08 ± 1.02 ^a^	2.75 ± 1.97 ^c^	0.001
Elasticity	79.91 ± 12.87 ^b^	74.24 ± 10.39 ^a^	74.05 ± 10.28 ^a^	72.23 ± 10.50 ^a^	0.001
Gumminess	9.98 ± 3.05 ^a^	12.87 ± 3.90 ^b^	15.72 ± 5.73 ^c^	19.57 ± 4.68 ^d^	0.001
L*(e)	76.92 ± 2.65 ^c^	67.58 ± 4.26 ^b^	64.03 ± 3.46 ^b^	59.37 ± 2.63 ^a^	0.001
L*(i)	87.84 ± 1.54 ^c^	86.65 ± 2.10 ^b^	84.58 ± 1.61 ^b^	79.41 ± 2.69 ^a^	0.001
chroma(e)	22.93 ± 3.36	24.27 ± 3.08	26.24 ± 3.51	26.16 ± 2.97	0.051
chroma(i)	12.34 ± 1.34 ^a^	14.05 ± 1.21 ^bc^	14.18 ± 1.05 ^c^	12.89 ± 0.88 ^ab^	0.001
hue angle(e)	91.15 ± 3.05 ^c^	86.86 ± 5.31 ^b^	82.62 ± 2.41 ^a^	80.60 ± 3.98 ^a^	0.001
hue angle(i)	99.50 ± 1.56	99.61 ± 1.67	99.64 ± 1.72	100.87 ± 3.05	0.390
a*(e)	−0.29 ± 0.15 ^a^	1.58 ± 1.25 ^ab^	3.21 ± 1.42 ^bc^	4.35 ± 1.99 ^c^	0.001
a*(i)	−2.05 ± 0.48	−2.33 ± 0.49	−2.32 ± 0.43	−2.34 ± 0.55	0.397
b*(e)	22.92 ± 3.37	24.13 ± 2.86	25.98 ± 3.43	25.74 ± 2.84	0.071
b*(i)	12.11 ± 1.29 ^a^	13.80 ± 1.17 ^bc^	13.87 ± 1.04 ^c^	12.55 ± 0.94 ^ab^	0.001

LSM: Least square mean; L*(e), chroma(e), hue angle(e), a*(e) and b*(e) correspond to parameters measured on cheese surface (external) and L*(i), chroma(i), hue angle(i), a*(i) and b*(i) correspond to parameters measured inside the cheese (internal); ^a–d^: Within a row, means marked with different superscripts differ significantly (*p* < 0.05).

**Table 2:** Sensorial texture, odour and flavour characteristics of Palmero cheese.

	Ripening Time (R)				
	15 d	30 d	60 d	90 d	
		LSM			Effect
Roughness	2.88 ± 0.28 ^a^	2.98 ± 0.30 ^a^	3.87 ± 0.32 ^b^	3.87 ± 0.36 ^b^	0.001
Superficial moisture	2.88 ± 0.99 ^c^	2.75 ± 1.07 ^c^	2.11 ± 0.25 ^b^	1.45 ± 1.71 ^a^	0.001
Elasticity	2.82 ± 0.98 ^b^	2.57 ± 1.03 ^ab^	2.10 ± 0.42 ^a^	2.18 ± 1.37 ^ab^	0.027
Firmness	3.75 ± 0.84	3.96 ± 1.15	4.04 ± 0.91	4.28 ± 0.72	0.203
Friability	4.11 ± 0.70 ^a^	4.23 ± 0.91 ^a^	4.92 ± 0.72 ^b^	4.61 ± 1.02 ^ab^	0.002
Adhesivity	4.30 ± 0.27 ^b^	4.30 ± 0.27 ^b^	3.34 ± 0.72 ^a^	3.46 ± 0.62 ^a^	0.001
Solubility	4.35 ± 0.75	4.08 ± 1.17	3.86 ± 0.62	4.01 ± 0.51	0.145
Mouth moisture	3.32 ± 0.37 ^c^	3.20 ± 0.50 ^c^	2.35 ± 0.69 ^b^	1.87 ± 0.34 ^a^	0.001
Granulosity	3.50 ± 0.38	3.55 ± 0.43	3.74 ± 0.83	3.63 ± 0.99	0.611
Acidity	2.22 ± 0.81 ^c^	1.88 ± 0.75 ^bc^	1.25 ± 0.40 ^a^	1.70 ± 0.60 ^ab^	0.001
Saltiness	3.75 ± 0.55 ^a^	3.94 ± 0.45 ^a^	4.28 ± 0.34 ^b^	4.32 ± 0.48 ^b^	0.001
Pungency	0.20 ± 0.37 ^a^	0.30 ± 0.55 ^a^	0.96 ± 0.78 ^b^	1.63 ± 1.14 ^c^	0.001
Bitterness	0.30 ± 0.55 ^b^	0.15 ± 0.27 ^ab^	0.00 ^a^	0.00 ^a^	0.001
Odour intensity	2.39 ± 0.63 ^a^	3.43 ± 1.15 ^b^	3.57 ± 1.16 ^b^	4.21 ± 0.91 ^b^	0.001
Flavour intensity	2.20 ± 0.71 ^a^	2.88 ± 0.72 ^b^	3.16 ± 0.83 ^b^	4.50 ± 0.64 ^c^	0.001

LSM: Least square mean; ^a-c^: Within a row, means marked with different superscripts differ significantly (*p* < 0.05).

**Table 3:** Correlation analysis of sensorial and instrumental texture characteristics with physicochemical parameters.

	Moisture	Protein	Fat	pH (External)	pH (Internal)
Fracturability	−0.578 **	0.413 **	0.452 **	0.603 ***	0.274, NS
Hardness	−0.504 **	0.343 *	0.358 *	0.601 ***	0.389 **
Cohesiveness	−0.178, NS	−0.056, NS	0.183, NS	0.215, NS	0.187, NS
Adhesiveness	−0.020, NS	0.067, NS	0.008, NS	0.097, NS	−0.008, NS
Elasticity	−0.163, NS	−0.228, NS	−0.230, NS	−0.179 NS	−0.153, NS
Gumminess	−0.352 *	0.105, NS	0.218, NS	0.438 **	0.285 *
Roughness	−0.778 ***	0.336 *	0.629 ***	0.696 ***	0.420 **
Sup. Moisture	0.593 **	−0.608 ***	−0.412 **	−0.750 ***	−0.513 **
Elasticity	0.513 **	−0.436 **	−0.229, NS	−0.587 **	−0.405 **
Firmness	−0.007, NS	−0.042, NS	−0.155, NS	0.057, NS	−0.079, NS
Friability	−0.206, NS	0.234, NS	−0.068, NS	0.317 *	0.170, NS
Adhesivity	0.377 **	−0.038, NS	−0.415 **	−0.402 **	−0.264, NS
Solubility	−0.135, NS	−0.112, NS	0.314 *	0.076, NS	0.068, NS
Mouth moisture	0.679 ***	−0.308 *	−0.461 **	−0.605 **	−0.353 *
Granulosity	−0.355 *	0.286 *	0.103, NS	0.343 *	0.289 *

*, **, ***: Significant at *p* < 0.05, 0.01 and 0.001, respectively; NS: not significant.

**Table 4:** Correlation analysis of sensorial terms and instrumental parameters for assessment of cheese texture.

	Fracturability	Hardness	Cohesiveness	Adhesiveness	Elasticity	Gumminess
Roughness	0.626 ***	0.633 ***	0.251 NS	0.150 NS	−0.116 NS	0.523 **
Superficial Moisture	−0.730 ***	−0.725 ***	−0.146 NS	−0.146 NS	0.336 *	−0.437 **
Elasticity	−0.603 **	−0.638 ***	−0.099 NS	−0.054 NS	0.089 NS	−0.472 **
Firmness	0.003 NS	0.058 NS	0.003 NS	0.157 NS	0.133 NS	0.137, NS
Friability	0.268 NS	0.318 *	−0.057 NS	0.074 NS	0.060 NS	0.242, NS
Adhesivity	−0.210 NS	−0.105 NS	−0.097 NS	−0.169 NS	0.088 NS	−0.023, NS
Solubility	0.064 NS	0.082 NS	0.192 NS	0.037 NS	−0.216 NS	0.023, NS
Mouth moisture	−0.688 ***	−0.709 ***	−0.158 NS	−0.037 NS	0.188 NS	−0.523 **
Granulosity	0.341 *	0.428 **	0.010 NS	−0.250 NS	−0.035 NS	0.345 *

*, **, ***: Significant at *p*<0.05, 0.01 and 0.001, respectively; NS: not significant.

**Table 5:** Physicochemical composition, texture and colour attributes of Palmero cheese (30 days) from 4 dairy plants.

	Dairy Plants				
	A	B	C	D	
		LSM			Effect
pH (external)	5.20 ± 0.17	5.06 ± 0.17	5.05 ± 0.20	5.23 ± 0.19	0.104
pH (internal)	5.07 ± 0.08 ^c^	4.92 ± 0.16 ^ab^	4.82 ± 0.10 ^a^	5.11 ± 0.14 ^c^	0.009
Moisture, %	33.75 ± 3.48	34.83 ± 4.51	35.30 ± 4.56	30.10 ± 3.83	0.244
Fat, % of TS	48.46 ± 2.08	47.45 ± 3.89	50.81 ± 2.11	53.54 ± 2.04	0.115
Protein, % of TS	33.14 ± 2.19	31.04 ± 1.65	30.30 ± 1.64	28.94 ± 1.70	0.435
Fracturability	123.03 ± 29.82 ^c^	90.60 ± 31.15 ^a^	77.96 ± 33.88 ^a^	107.13 ± 31.60 ^b^	0.001
Hardness	162.51 ± 64.40	164.42 ± 60.69	172.32 ± 62.04	152.65 ± 52.62	0.349
Cohesiveness	0.09 ± 0.02 ^a^	0.12 ± 0.02 ^b^	0.14 ± 0.02 ^c^	0.09 ± 0.01 ^a^	0.001
Adhesiveness	0.92 ± 0.87 ^a^	2.91 ± 1.66 ^b^	1.15 ± 1.01 ^a^	2.52 ± 2.01 ^b^	0.001
Elasticity	81.18 ± 13.74 ^b^	66.96 ± 7.80 ^a^	65.34 ± 7.94 ^a^	84.29 ± 7.98 ^b^	0.001
Gumminess	11.30 ± 6.71 ^a^	12.99 ± 5.11 ^ab^	15.33 ± 5.30 ^b^	11.68 ± 4.83 ^a^	0.007
L*(e)	62.63 ± 7.14	71.16 ± 8.80	68.61 ± 7.93	67.68 ± 5.26	0.065
L*(i)	87.33 ± 2.50	88.22 ± 3.97	86.91 ± 4.53	83.92 ± 3.82	0.051
chroma(e)	23.47 ± 1.32 ^a^	21.93 ± 3.09 ^a^	28.73 ± 3.12 ^b^	22.85 ± 1.98 ^a^	0.003
chroma(i)	14.27 ± 1.14 ^bc^	12.56 ± 1.03 ^a^	13.62 ± 1.16 ^ab^	15.43 ± 0.89 ^c^	0.003
hue angle(e)	90.12 ± 2.87 ^b^	88.16 ± 7.38 ^b^	79.23 ± 4.76 ^a^	89.78 ± 4.56 ^b^	0.007
hue angle(i)	100.59 ± 0.96 ^c^	97.31 ± 0.88 ^a^	101.33 ± 2.18 ^c^	99.04 ± 1.05 ^b^	0.001
a*(e)	0.02 ± 0.97 ^a^	0.71 ± 2.97 ^a^	5.42 ± 2.45 ^b^	0.15 ± 1.96 ^a^	0.003
a*(i)	−2.63 ± 0.31 ^a^	−1.60 ± 0.24 ^b^	−2.69 ± 0.44 ^a^	−2.42 ± 0.29 ^a^	0.001
b*(e)	23.45 ± 1.30 ^a^	21.86 ± 2.99 ^a^	28.17 ± 3.00 ^b^	22.81 ± 1.87 ^a^	0.004
b*(i)	14.02 ± 1.13 ^ab^	12.45 ± 1.02 ^a^	13.35 ± 1.17 ^a^	15.23 ± 0.88 ^b^	0.003

LSM: Least square mean; L*(e), chroma (e), hue angle(e), a*(e) and b*(e) correspond to parameters measured on cheese surface (external) and L*(i), chroma(i), hue angle(i), a*(i) and b*(i) correspond to parameters measured inside the cheese (internal); ^a–d^: Within a row, means marked with different superscripts differ significantly (*p* < 0.05).

**Table 6:** Sensorial texture, odour and flavour characteristics of Palmero cheese (30 days) from 4 dairy plants.

	Dairy Plants				
	A	B	C	D	
		LSM			Effect
Roughness	3.21 ± 0.58 ^b^	3.03 ± 0.53 ^ab^	2.75 ± 0.69 ^a^	2.92 ± 0.39 ^ab^	0.018
Superficial moisture	2.00 ± 0.43 ^a^	2.50 ± 0.49 ^b^	4.50 ± 1.44 ^c^	2.00 ± 0.36 ^a^	0.001
Elasticity	2.00 ± 0.77 ^a^	4.29 ± 1.14 ^b^	2.00 ± 1.09 ^a^	2.00 ± 0.35 ^a^	0.001
Firmness	5.64 ± 0.78 ^c^	3.14 ± 0.76 ^a^	2.86 ± 0.96 ^a^	4.21 ± 0.48 ^b^	0.001
Friability	5.71 ± 0.28 ^b^	3.71 ± 0.72 ^a^	3.79 ± 0.89 ^a^	3.71 ± 0.53 ^a^	0.001
Adhesivity	4.21 ± 0.25	4.29 ± 0.71	4.50 ± 0.73	4.21 ± 0.68	0.141
Solubility	2.21 ± 0.50 ^a^	4.36 ± 0.29 ^b^	5.25 ± 0.54 ^c^	4.50 ± 0.43 ^b^	0.001
Mouth moisture	2.64 ± 0.79 ^a^	3.14 ± 0.64 ^b^	3.79 ± 0.81 ^c^	3.21 ± 0.50 ^b^	0.001
Granulosity	4.00 ± 0.67 ^a^	3.14 ± 0.40 ^b^	3.79 ± 0.43 ^b^	3.29 ± 0.28 ^a^	0.001
Acidity	1.29 ± 0.34 ^a^	1.29 ± 0.53 ^a^	2.99 ± 1.11 ^c^	1.96 ± 0.52 ^b^	0.001
Saltiness	4.21 ± 0.39 ^b^	4.11 ± 0.16 ^b^	4.00 ± 0.69 ^b^	3.43 ± 0.49 ^a^	0.001
Pungency	1.21 ± 0.93 ^b^	0.00 ^a^	0.00 ^a^	0.00 ^a^	0.001
Bitterness	0.00 ^a^	0.00 ^a^	0.00 ^a^	0.61 ± 0.53 ^b^	0.001
Odour intensity	4.71 ± 0.97 ^b^	2.57 ± 0.79 ^a^	2.71 ± 0.71 ^a^	2.35 ± 1.94 ^a^	0.001
Flavour intensity	3.79 ± 1.27 ^c^	2.29 ± 1.16 ^a^	3.21 ± 0.63 ^b^	2.21 ± 0.82 ^a^	0.001

LSM: Least square mean; ^a–c^: Within a row, means marked with different superscripts differ significantly (*p* < 0.05).

**Table 7:** Factor matrix obtained after a Varimax rotation.

	Factor			
% Cumulative Variance	1	2	3	4
Roughness	0.170	−0.327	0.169	−0.738
Superficial moisture	0.030	0.923	0.270	0.170
Elasticity	−0.698	−0.286	0.617	−0.066
Firmness	0.661	−0.653	−0.237	−0.153
Friability	0.812	−0.434	0.141	−0.230
Adhesivity	0.048	0.233	0.206	0.825
Solubility	−0.633	0.715	−0.125	0.212
Mouth moisture	−0.273	0.772	−0.014	0.246
Granulosity	0.857	0.098	0.127	0.339
Odour intensity	0.837	−0.418	0.292	−0.031
Flavour intensity	0.906	0.125	0.269	−0.095
Acidity	0.065	0.915	−0.245	0.205
Saltiness	0.300	−0.130	0.737	0.368
Pungency	0.811	−0.487	0.165	−0.144
Bitterness	−0.293	−0.196	−0.897	0.136

**Table 8:** Stepwise discriminant analysis of cheese samples according to ripening period, numbers and (%).

	Ripening Period	Predicted Group				Total
		1	2	3	4	
Initial Group	15 d	26 (92.9)	2 (7.1)	0.0	0.0	100.0
	30 d	5 (17.9)	23 (82.1)	0.0	0.0	100.0
	60 d	0.0	0.0	28 (100.0)	0.0	100.0
	90 d	0.0	0.0	0.0	28 (100.0)	100.0

93.8% samples well classified.

## Data Availability

The data are not publicly available because they were obtained as part of the Canary Islands Government’s (DOQUECAN) and currently belong to the Regulatory Council of the Palmero Cheese Protected Designation of Origin. The data presented in this study may be available on request from the corresponding author.
